# Evolutionary conservation analysis of human sphingomyelin metabolism pathway genes

**DOI:** 10.1016/j.heliyon.2024.e40810

**Published:** 2024-11-29

**Authors:** Siyuan Wang, Huan Jiang, Moran Hu, Yingyun Gong, Hongwen Zhou

**Affiliations:** Department of Endocrinology and Metabolism, the First Affiliated Hospital of Nanjing Medical University, 210029, Nanjing, China

**Keywords:** Sphingomyelin, Ceramide, *de novo* synthesis pathway, Salvage synthesis pathway, Interspecies conservation status

## Abstract

Sphingomyelin is an important member of the sphingolipid family and was first reported more than a century ago. It has been demonstrated that sphingomyelin plays a crucial role in compositing cell membranes and signaling pathways. Despite extensive functional studies on the sphingolipid metabolism pathway genes, one intriguing question remains: how does the emergence of these genes during evolution correlate with the acquisition of new functions in different species? By employing an evolutionary conservation analysis, the sequence of occurrence of biological processes during evolutionary history can be elucidated. Here we summarize and analyze the conservation status of the genes involved in sphingomyelin metabolism.

## Abbreviations

ACER1: alkaline ceramidase 1
ACER2: alkaline ceramidase 2
ARSA: arylsulfatase A
ASAH1: N-acylsphingosine amidohydrolase 1
ASAH2: N-acylsphingosine amidohydrolase 2
B4GALT5: beta-1,4-galactosyltransferase 5
B4GALT6: beta-1,4-galactosyltransferase 6
CERK: ceramide kinase
CERS1: ceramide synthase 1
CERS2: ceramide synthase 2
CERS3: ceramide synthase 3
CERS4: ceramide synthase 4
CERS5: ceramide synthase 5
CERS6: ceramide synthase 6
DEGS1: delta 4-desaturase, sphingolipid 1
DEGS2: delta 4-desaturase, sphingolipid 2
ENPP7: ectonucleotide pyrophosphatase/phosphodiesterase 7
GALC: galactosylceramidase
GalCer: galactosylceramide
GBA1: glucosylceramidase beta 1
GBA2: glucosylceramidase beta 2
GLA: galactosidase alpha
GLB1: galactosidase beta 1
GlcCer: glucosylceramide
GO: Gene Ontology
HEXA: hexosaminidase subunit alpha
HEXB: hexosaminidase subunit beta
KDSR: 3-dehydrosphinganine reductase
NEU1: neuraminidase 1
NEU2: neuraminidase 2
NEU3: neuraminidase 3
NEU4: neuraminidase 4
SGMS1: sphingomyelin synthase 1
SGMS2: sphingomyelin synthase 2
SGPP1: sphingosine-1-phosphate phosphatase 1
SGPP2: sphingosine-1-phosphate phosphatase 2
SMase: sphingomyelinase
SMPD1: sphingomyelin phosphodiesterase 1
SMPD2: sphingomyelin phosphodiesterase 2
SMPD3: sphingomyelin phosphodiesterase 3
SMPD4: sphingomyelin phosphodiesterase 4
SPHK1: sphingosine kinase 1
SPHK2: sphingosine kinase 2
SPTLC1: serine palmitoyltransferase long chain base subunit 1
SPTLC2: serine palmitoyltransferase long chain base subunit 2
SPTLC3: serine palmitoyltransferase long chain base subunit 3
UGCG: UDP-glucose ceramide glucosyltransferase
UGT8: UDP glycosyltransferase 8

## 1 Introduction

Sphingomyelin, a crucial component of eukaryotic cell membranes, is distinguished from other phospholipids by its sphingosine backbone instead of a glycerol group. This unique structure endows sphingomyelin with specific properties that are vital for cellular functions. Sphingomyelin is mainly present in the cell membrane of animal cells, accounting for about 23 % of total membrane lipids [1]. [2], and is also distributed in Golgi apparatus and lysosomes. It is a type of phospholipid characterized by a sphingoid long-chain base, amide-linked fatty acid, and a phosphorylcholine head group. Upon hydrolysis, sphingomyelin yields a fatty acid, sphingosine, phosphate, and choline. The fatty acid is typically saturated, contributing to the rigidity and stability of the cell membrane [3]. Sphingomyelin is a very high proportion of phospholipids found in most tissues of mammals, especially in the myelin sheath of neurons (around the nerve axon) [4]. Moreover, the distribution and subtype of sphingolipids in foodstuffs do not exhibit uniformity. Sphingomyelin is primarily distributed in animal products, including aquatic products, meat products, eggs, dairy products, and so forth. Dietary sphingomyelin is primarily hydrolyzed by alkylphospholipase A2 (Alk SMase) in the intestinal mucosa into ceramide and choline phosphate. The absorption of these metabolites can influence the metabolic level of cholesterol and triglycerides in the body. It has been reported that dietary sphingomyelin in the colon can reduce the occurrence and development of aortic plaques [5], which helps to treat and prevent obesity [6]^.^ [7]. The addition of sphingomyelin to a variety of food products, including dairy products, infant formula, nutritional supplements, pet food, and other diets, has become a common practice [8].

The principal constituents of lipid rafts are phospholipids, cholesterol, and proteins. From a molecular perspective, cholesterol lacks the extended fatty acid chains observed in other sterols. However, its structural similarity to sterols allows for facile insertion into the long fatty acid chains of phospholipid molecules. In particular, sphingomyelin molecules typically possess long chains of saturated fatty acids, and cholesterol and sphingomyelin molecules are prone to spontaneous formation of tightly aggregated and fluidic ordered lipid molecular domains [9]. However, due to the limitations of the available observation methods, the precise interaction mechanism between cholesterol and sphingomyelin molecules on the cell membrane remains unclear. Nevertheless, a recent study utilized ostreolysin A and indicated that the interaction between sphingomyelin and cholesterol may alter the intrinsic conformation of sphingomyelin, thereby facilitating the chelation of cholesterol on the membrane and the formation of a lipid raft-specific domain [9], involved in cell signaling, lipid and protein sorting, and membrane transport [10]. Sphingomyelin is interconverted with a variety of bioactive sphingolipids, such as ceramide and sphingosine 1-phosphate. These bioactive lipids also act on specific targets within cells, regulate various signal transduction pathways, and play key roles in multiple disease models [11].

Sphingomyelin is particularly abundant in the myelin sheath that surrounds and insulates nerve cells, contributing to the proper functioning of the nervous system. Additionally, it is involved in regulating membrane fluidity and participating in cell signaling processes [12]. Sphingomyelin's structural and functional significance makes it a key component of cellular membranes and an important player in various physiological processes [13].

Abnormalities in sphingomyelin metabolism may lead to various diseases, such as neurological degeneration [14], cardiovascular disease [15]^,^ and metabolic diseases. Although sphingomyelin metabolic pathway genes have been extensively studied, what new functions can these genes appear between different species during evolution? Many reviews provide detailed evidence regarding sphingolipids from different aspects [16]^.^ [17]^.^ [18], as well as some user-friendly databases [19] and websites [20], but scarce information is focused on evolution analysis. We systematically analyzed the sequence conservation of human sphingomyelin metabolic pathway genes to address this question.

Performing evolutionary conservation analysis of the sphingolipid pathway can provide insights into why this pathway is related to metabolic disorders and human health for several reasons. Firstly, identify key genes and enzymes that are essential for the pathway's function. Secondly, understand how disruptions in sphingolipid metabolism contribute to various metabolic disorders and develop potential therapeutic choices for related diseases. Thirdly, provide clues about the functional importance of different components within the family.

## 2 Methods

### 2.1 Data collection

The human sphingomyelin metabolism pathway was constructed according to the sphingolipid metabolism map in the KEGG pathway database. Genes involved in the sphingolipid metabolism pathway were also collected from the KEGG database (https://www.kegg.jp/entry/map00600).

### 2.2 Gene conservation analysis

Gene conservation analysis was performed using information provided by the HomoloGene database. The species conservation data for sphingomyelin metabolic pathway genes were retrieved from the HomoloGene database using gene symbols as queries. The evolutionary conservation status of each gene was further verified using the Protein BLAST database, with E-value <1^e−6 and percent identity >30 % as the threshold for homology gene identification, and the maximum number of aligned sequences to display was set as 5000. The Protein BLAST analysis results were in agreement with the sequence conservation information in the HomoloGene database for all analyzed genes.

### 2.3 Gene Ontology analysis

Gene Ontology (GO) analysis was performed with clusterProfiler package in R and the online Metascape software (https://metascape.org/gp/index.html#/main/step1). Raw p-value was adjusted by the Benjamini-Hochberg method, using q-value <0.05 as the threshold to select enriched GO terms. Only GO terms with more than 3 input genes were considered.

### 2.4 Functional analysis of sphingomyelin metabolism related pathway genes

The known functions of sphingomyelin metabolism related pathway genes were collected from the GeneCards database (https://www.genecards.org), UniProt (https://www.uniprot.org), and thorough literature searches, the neofunctionalization roles of each gene were inferred by combining its function and evolutionary conservation information.

### 2.5 Data availability

The list of genes is shown in Table S1.

## 3 Results

**Fig. 1:**
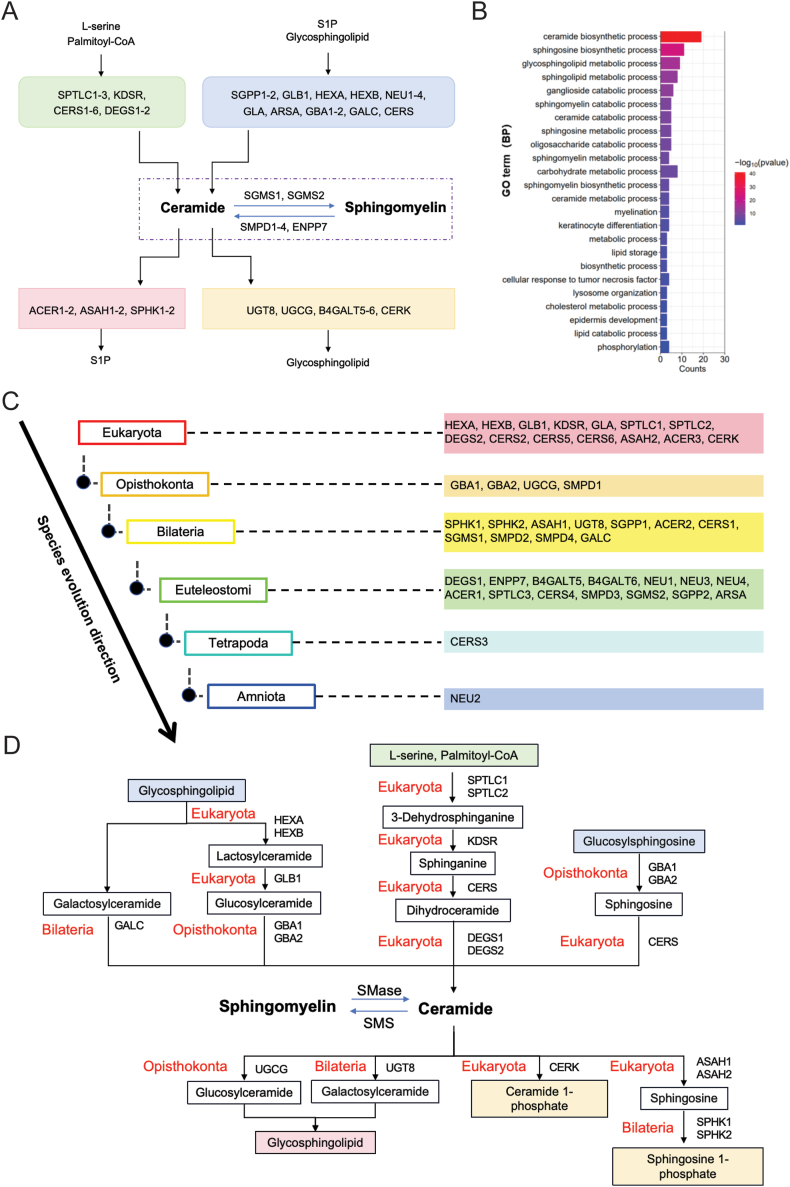
Sphingolipid pathway and conservation status of related genes. A. List of genes involved in the sphingolipid metabolism. B. Gene ontology (GO) analysis of genes in sphingolipid metabolism. The enriched GO terms in the biological process category are shown, and the X-axis represents the number of genes in each GO term. C. The conserved status of sphingolipid metabolism pathway genes during evolution. Genes are displayed on the corresponding evolutionary branch, where sequence conservation is first observed. D. The major metabolites of the SM metabolic pathway and the conserved status of genes in this pathway.

Sphingomyelin is synthesized by the key enzyme, sphingomyelin synthase (SMS), by transferring phosphocholine from phosphatidylcholine to ceramide. Ceramide is a key signaling molecule in cellular oxidative stress pathway and plays an important role in membrane homeostasis, affecting membrane microdomain function, membrane vesicle formation, fusion, fission, and vesicle transport, which are involved in many pathophysiological processes [21]. There are three main ways of generating ceramide. The *de novo* synthesis pathway of L-serine and Palmitoyl-CoA is best known, and several essential enzymes contribute to its synthesis, including serine palmitoyl transferase (SPT), 3-ketosphinganine reductase (KDSR), dihydroceramide desaturase (DEGS) and ceramide synthetase (CERS). The salvage pathway is another crucial pathway that facilitates glycosphingolipid and sphingosine fill ceramide pool through the action of glycosidases and CERS mainly in late endosomes and lysosomes. In addition, ceramide also orients from sphingomyelin hydrolysis via sphingomyelinase (SP). Furthermore, the homeostasis of ceramide is modulated by a few enzymes regulating S1P and GSLs synthesis (Fig. 1A). GO analysis showed that the genes of sphingolipid pathways are enriched in various sphingolipid biosynthetic, catabolic, and metabolic processes, playing key roles in the lipid storage, cellular responses, and related biological regulations (Fig. 1B).

The normal metabolism of sphingomyelin plays a significant role in the functioning of the human body. When the genes encoding the enzymes involved in the metabolic pathway are mutated, the resulting abnormalities in the metabolism of sphingomyelin and its metabolites can lead to the development of related diseases. For example, SMS is a key enzyme in the sphingomyelin synthesis pathway. Mutations in the SMS1 gene have been linked to progressive hearing loss [22]. The etiology of osteoporosis and skeletal dysplasia is attributed to pathogenic variation in the SMS2 gene [23]. Furthermore, sphingomyelin is a crucial element in the metabolism and thermogenesis of adipocytes. SPTLC represents the initial enzyme and is among the most pivotal enzymes within the cascade reaction. The systemic and adipose-tissue-specific deletion of SPT prevents high-fat diet (HFD)-induced adipocyte hypertrophy, alters the profile of infiltrating macrophages, and increases the number of brown/beige adipocytes. Enhanced mitochondrial activity and accelerated glucose uptake are relevant for the prevention and treatment of obesity [24]. Therefore, an understanding of the sequence conservation of genes in sphingomyelin metabolic pathways facilitates a more comprehensive grasp of the evolutionary forces that shape gene sequence and the functions of diverse members of sphingomyelin metabolic pathways. This, in turn, offers more precise targets for the treatment of diseases associated with sphingomyelin metabolism.

Over forty genes are involved in sphingolipid pathways (Table S1 and Fig. 1A). The ceramide de novo synthesis pathway contains 12 genes, and the salvage pathway contains another 14 genes. Sphingomyelin synthesis and hydrolysis processes include two and five genes respectively. Other metabolic pathways contain 11 genes. According to conservative state analysis, the de novo synthesis pathway genes are mainly conserved in Eukaryotes, and others in the salvage or metabolic pathways are mainly conserved in Bilateria and Euteleostomi (Fig. 1C and D).

**Fig. 2:**
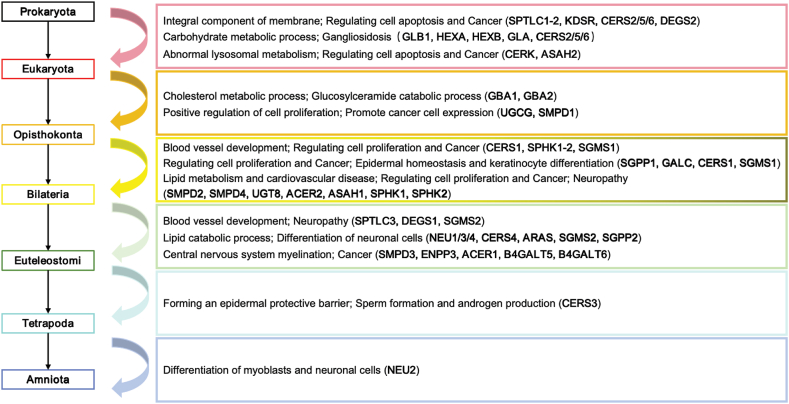
Conservation status of sphingolipid metabolism pathway genes. Review of the conservation status of sphingolipid metabolism pathway genes and their potential functions during evolution.

**Fig. 3:**
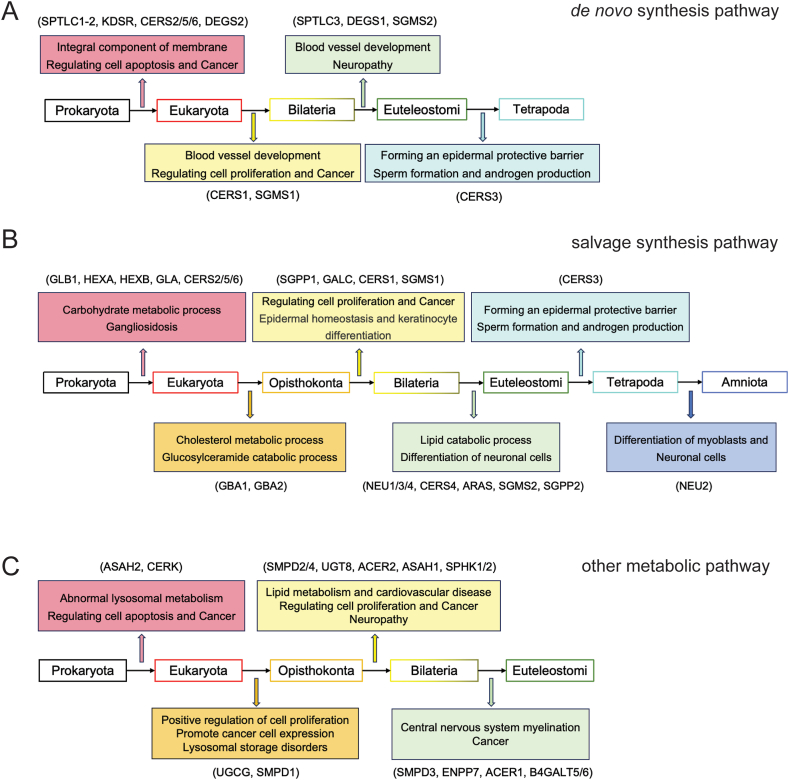
Detailed conservation status of three sphingolipid related pathways. The function of de novo synthesis pathway (A), salvage synthesis pathway (B), and other metabolic pathway (C) genes to drive new functionalization during evolution. The boxes above the arrows represent functions that evolved with the emergence of evolutionary branches, which are outlined by the same color. Information on the emergence of evolutionary functions shown is inferred from the literature as well as from the GO database.

Most of the de novo pathway enzymes are conserved from Euteleostomi to humans and play an important role in membrane lipid biosynthesis. SPT is the most upstream key enzyme in the de novo synthesis pathway, and functions as the rate-limiting enzyme by converting L-serine and Palmitoyl-CoA to 3-dehydrosphinganine together with pyridoxal 5′-phosphate. It binds with palmitoyl CoA and subsequently causes conformational changes, and their interaction is crucial for the dynamic regulation of de novo synthesis of sphingolipids [25]. SPT holozyme is a heterodimer composed of two core subunits SPT long chain base subunit 1 and 2 (SPTLC1 and SPTLC2) with 20 % sequence homology. SPTLC2 and SPT long chain base subunit 3 (SPTLC3) are homologous subunits with 68 % sequence consistency, and this complex is mainly embedded in the endoplasmic reticulum membrane [26]. SPT is widely expressed in vivo and has been found in brain, lung, liver, kidney and muscle. Among them, SPTLC1 and SPTLC2 are conserved in Eukaryotes (Fig. 2, Fig. 3A), and they are highly expressed in pyramidal neurons, colon epithelium, and mucosal macrophages in the brain [27]. Mutations in this gene have been reported in patients with inherited sensory neuropathy type 1 [28], tumor growth [29], and alternative splicing variants encoding different isoforms have been identified. SPTLC3 is only expressed in certain specific tissues, such as the placenta, skin, and certain glands [30]. SPTLC3 is only conserved in Euteleostomi, and it has been reported playing an important role in cardiovascular diseases [31] and neuropathy [32]. CERS is one of the key enzymes in both de novo and salvage synthesis pathways, and there are six subtypes (CERS1-6), all located in the endoplasmic reticulum. Each synthetase has a different acyl-CoA carbon chain length preference, and acyl-chain specificity is determined by the sequence of eleven residues in a ring between the last two transmembrane domains. The length, unsaturated number, and location of the fatty acid contribute to the function of different ceramide subtypes. Natural ceramides can be divided into long chain ceramides (C14:0-C20:0; LCC) and ultra-long chain ceramide (C22:0-C26:0; VLCC). Short chains (C2-C8; SCC) ceramides are not naturally synthesized and are often used in exogenous drug delivery studies, playing an important role in anti-tumor and other aspects [33]. The first three CERS subtypes appear, which are conserved in prokaryota, CERS2 is widely distributed in tissues and has the highest expression level in the kidneys and liver. CERS5 is mainly distributed in lung epithelium, skeletal muscle and kidney, while CERS6 is mainly distributed in brain and kidney. Their high expression in tissues plays an important role in regulating membrane composition, influencing cell activity and apoptosis [34]. CERS3 appeared at the latest and was conserved only in Tetrapoda (Fig. 2, Fig. 3A). In addition, it mainly plays an important role in forming an epidermal protective barrier. Only CERS3 is mainly expressed in germ cells and plays a role in sperm formation and androgen production [35].

The salvage synthesis pathway is also an important source for the ceramide pool, which first converts complex sphingolipids into glycosphingolipid and sphingosine (Fig. 1D), and then generates ceramide through a re-acylation reaction, mainly in lysosomes and cell endosomes. In 2014, Ferrara et al. first discovered the β-hexosaminidase (HEX) derived from archaea, which is mainly composed of two subtypes in mammalian cells: HEXA and HEXB, encoding alpha and beta subunits respectively [36]. HEXA and HEXB are upstream enzymes that are conserved in Eukaryota (Fig. 1C and D). HEX is an important lysosomal enzyme and play important roles in lipid storage, myelination, and neuromuscular cell development, mediating the breakdown of GM2 gangliosides. The loss of its function leads to the accumulation of GM2 and progressive neurodegenerative diseases [37]. Although HEX is widely distributed in nature, most of the enzymes used for glycoside synthesis come from fungi [38]. Glucosylceramidase beta 1 and 2 (GBA1 and GBA2) isoforms are consistently conserved in Opisthokonta (Fig. 2), which cleave the β-glycosiside bond of glucosylceramide (GlcCer). In addition to its role in GlcCer catabolism, GBA1 and GBA2 also play an important role in cholesterol metabolism. These two GBA proteins do not have structural or sequence homology, but have similar enzymatic activity. Loss of function mutations in GBA1 leads to the accumulation of glucocerebroside, causing related lysosomal storage disease [39], while non lysosomal GBA2 can lead to the accumulation of GlcCer in various tissues outside the lysosome, such as the testes and liver [40]. The human neuraminidase family (hNEUs), also known as sialoglycans, is a family of exosanase enzymes that hydrolyze the terminal sialic acid residues of glycolipids and glycoproteins and are involved in important signaling pathways that have been implicated in a variety of diseases, including neurodegenerative diseases [41], cancer, infectious diseases, and cardiovascular disease [42]. Therefore, hNEUs is an important potential source of therapeutic drugs. There are four subtypes of hNEUs: NEU1, NEU3 and NEU4 are conserved in Euteleostomi (Fig. 2, Fig. 3B), while NEU2 appears late and is conserved only in Amniota (Fig. 2, Fig. 3B). The hNEUs play crucial physiological roles in regulating immunity responses, apoptosis, and differentiation in myoblasts and neuronal cells. Notably, the synthesis pathway of ganglioside appeared later than other sphingolipids.

SMS is the key enzyme catalyzing phosphatidylcholine and Ceramide to produce sphingomyelin and diacylglycerol by transferring phosphocholine from phosphatidylcholine to Ceramide. The SMS family consists of three members, SGMS1, SGMS2, and SGMSr (Sphingomyelin synthase1 and 2, Sphingomyelin synthase-related), but only the two isoenzymes SGMS1 and SGMS2 have catalytic activity [43]^.^ [44]. SGMS1 is mainly expressed in the Golgi apparatus and conserved in Bilateria (Fig. 2), located on human chromosome 11 and mouse chromosome 19, while SGMS2 is mainly present in the plasma membrane and Golgi apparatus, conserved in Euteleostomi (Fig. 2), located on human chromosome 4 and mouse chromosome 3 [45]. Although both SGMS1 and SGMS2 contain 6 transmembrane domains, SGMS1 has a sterile alpha motif at the N-terminus [46]. while SGMS2 is mainly present in the plasma membrane and Golgi apparatus, conserved in Euteleostomi (Fig. 2). Hydrolysis sphingomyelin back to ceramide by transferring of phosphocholine to DAG, facilitated by the action of SMase. This hydrolase is important in cell differentiation, immune and inflammatory responses, and intracellular cholesterol transport and metabolism. The activity of both SMS and SMase modulates the composition of the plasma membrane and regulates the contents of sphingomyelin and other sphingolipids. The homeostasis affects cell migration, apoptosis, and DAG-dependent signaling pathways, closely associated with inflammatory diseases, including atherosclerosis, hepatitis, and type 2 diabetes. SMase family members are classified primarily according to cationic dependence and pH of action, differing in characteristics, regulation, tissue distribution, and subcellular localization. The activation of SMase is mainly in lysosomes and plasma membranes. Moreover, Alk-SMase does not share any structural similarity with NSMases or ASMases. SMPD1 is an acid sphingomyelinase, conserved in Opisthokonta (Fig. 2, Fig. 3C). The latest discovery of the crystal structure of mammalian ASMase, with N-terminal saposin domain and catalytic domain, has been reported that the secretion form of ASMase depends on Zn2+ [47]. It has been reported that disease-causing mutations in the SMPD1 lead to abnormal ASMase activity, resulting in lysosomal storage disorders, such as Niemann-Pick disease. The three subtypes of SMPD2, SMPD3 and SMPD4 belong to neutral sphingomyelinase, among which SMPD2 and SMPD4 are generally expressed in various tissues and conserved in Bilateria (Fig. 2), while SMPD3 is mainly expressed in neurons of the central nervous system, closely related to the formation of myelin sheath in the central nervous system, and is conserved in Euteleostomi (Fig. 2, Fig. 3C). SMPD2 is mainly distributed in the endoplasmic reticulum and Golgi apparatus, while SMPD3 is mainly present in the plasma membrane and nuclear membrane, both of which are Mg2+- dependent. Ectonucleotide pyrophosphatase (ENPP7) is an alkaline sphingomyelinase, mainly distributed in the plasma membrane and lysosomes, highly expressed in the intestine, playing a crucial role in intestinal digestion, and to some extent located in the liver [48]. ENPP7 also degrades and inactivates platelet activating factor, so it can protect the intestinal mucosa from inflammation and tumorigenesis.

Similarly, ceramidase is classified according to its responsive pH ranges, including alkaline and acid ceramidase. Ceramidase hydrolyzes the connection between fatty acids in ceramide and sphingosine, widely distributed in animal tissues such as brain, kidney, and spleen. Alkaline ceramidase 1 and 2 (ACER1 and ACER2) belong to the alkaline ceramidase group conformed to Euteleostomi and Bilateria (Fig. 2, Fig. 3C), respectively. ACER1 appears the latest and is conserved in Euteleostomi, mainly distributed in the endoplasmic reticulum and highly expressed in the skin, while ACER2 is mainly distributed in the Golgi complex and highly expressed in the placenta, both of which play important roles in cell differentiation and apoptosis [49]^.^ [50]. N-acylsphingosine amidohydrolase 1 (ASAH1) belongs to the acidic ceramidase group conformed to Bilateria and is mainly distributed in lysosomes. It is a heterodimer composed of alpha (13 kDa) and beta (40 kDa) subunits [51] and N-acylsphingosine amidohydrolase 2 (ASAH2) belongs to the neutral ceramidase group, which appeared earliest conformed to Eukaryota (Fig. 2, Fig. 3C). Mainly located on the plasma membrane, expressed in the small intestine and colon, involved in the body's digestive process, and related to the occurrence of colon cancer [52].

Sphingosine kinase (SPHK) is a downstream lipid enzyme that catalyzes sphingosine phosphorylation to generate S1P. Two subtypes of SPHK1 and SPHK2 have been found in mammals, conserved in Bilateria (Fig. 2, Fig. 3C). SPHK1 is mainly found in the cytoplasm and plasma membrane of red blood cells, endothelial cells, and mast cells. SPHK2 is large and localized in the endoplasmic reticulum, nucleus, and mitochondria. Both subtypes are involved in brain and blood vessel development and cell proliferation. In mice and rats, inhibition of sphingolipids de novo synthase prevents the development of diabetes, atherosclerosis, hypertension, and heart failure [53]. Activation of DAG-PKCε pathway is also associated with hepatic insulin resistance in most diseases such as obesity, diabetes, and non-alcoholic fatty liver disease [54]. Sphingosine, the metabolite of ceramide, is associated with cell apoptosis and can promote tumor growth, neovasculation, and inflammation, so it can become an important target for cancer treatment [55]. GlcCer and galactosylceramide (GalCer) are important molecules found in animals, plants, and fungi, and play an important role in neurodegenerative and neoplastic diseases [56].

## 4 Discussion

The metabolic pathway of sphingomyelin plays an important role in many physiological processes and drug metabolism. Here, we systematically analyzed the sequence conservation of genes in sphingomyelin metabolism pathway and found the link between the genes and the acquisition of new functions during the evolution of different species. For example, the appearance of the sphingomyelin synthesis and hydrolysis pathways, as well as the conservation of the corresponding enzymes are well correlated with membrane composition in eukaryotes, cell proliferation, epidermal homeostasis, and keratinocyte differentiation in Bilateria, nerve cell differentiation and the formation of the central nervous system in Euteleostomi. CERS2, 5, and 6 are conserved in Eukaryota and are widely expressed in various tissues. They are responsible for the synthesis of ceramides in multiple tissues and are sufficient to maintain the most basic biological functions of the body. CERS2 was the highest expressed in liver tissue, CERS5 was mainly expressed in the endometrium and placenta, and CERS6 was mainly expressed in the colon and thyroid. With the change of species, the distribution and expression of other CERS subtypes are less than those of the above subtypes. CERS1 is conserved in Bilateria and mainly expressed only in brain tissue, where it is responsible for ceramide synthesis processes, and a general deficiency of CERS1 in mice leads to cerebellar lobe defects, progressive atrophy, and neuronal apoptosis. CERS4 is conserved in Euteleostomi and is mainly highly expressed in the thyroid and prostate. However, CERS3 was finally conserved in Tetrapoda and was only expressed in the skin, esophagus, and testis.

SMPD1 is conserved in Opisthokonta, which is the earliest SMase subtype. It is widely expressed in various tissues with high content, especially in the kidney and thyroid, which is enough to maintain the basic physiological balance of the body. SMPD2 and SMPD4 are conserved in Bilateria, with SMPD2 mainly expressed in testicular tissue and SMPD4 expressed in bone marrow, testes, and thyroid. They are also commonly expressed in various tissues, but their content is significantly lower than SMPD1; SMPD3 and ENPP7 are conserved in Euteleostomi, and they are the last conserved SMase subtypes, and their expression is lower in various tissues. Especially, ENPP7 is almost only expressed in the duodenum and small intestine, so it is closely related to digestive system diseases. Similarly, SPTLC1 and SPTLC2 were the first to be conserved in Eukaryota, and they were generally highly expressed in various tissues, sufficient for normal biological functions of the body.

Furthermore, the conservation of these genes differs across various models. For example, sphingomyelin plays an important role in regulating the diurnal behavior and lifespan of fruit flies. In comparison to humans, the metabolic genes involved in the rescue pathway of sphingomyelin in fruit flies exhibit relatively lower levels of conservation. These include genes such as NEU, B4GALT5, B4GALT6, GLAC, GLA, UGT8, and others. SMS represents a pivotal enzyme that facilitates the transfer of phosphatidylcholine and ceramide from phosphatidylcholine to ceramide, resulting in the production of sphingomyelin and diacylglycerol. Nevertheless, in the fruit fly, the SMS1 and SMS2 genes are not conserved.

As species evolve, each enzyme subtype becomes more complete in distribution and division of labor. The first conserved enzyme subtypes are generally highly expressed in various tissues. With the emergence of other subtypes of conservatism, they collaborate to be responsible for the synthesis of sphingomyelin in various tissues of the body, ensuring a more complete and orderly biological metabolic mechanism. Moreover, these lead to a better understanding of the drivers of gene sequence evolution and the functions of different members in the sphingolipid metabolism pathway and provide more precise targets for the treatment of diseases related to sphingomyelin metabolism.

## 5 Research limitations

The potential role of newly functionalized sphingomyelin pathway genes in evolution revealed in this study was inferred from species conservation information and literature searches, and further experimental studies are needed to support these speculations.

## CRediT authorship contribution statement

**Siyuan Wang:** Writing – original draft, Visualization, Methodology, Formal analysis, Data curation. **Huan Jiang:** Writing – review & editing, Visualization, Validation. **Moran Hu:** Writing – review & editing, Visualization, Validation. **Yingyun Gong:** Writing – review & editing, Visualization, Validation, Supervision, Investigation, Funding acquisition, Conceptualization. **Hongwen Zhou:** Writing – review & editing, Supervision, Funding acquisition, Conceptualization.

## Ethics approval and consent to participate

Not applicable.

## Consent for publication

All authors approved the manuscript and give consent for submission and publication.

## Availability of data and materials

Data availability is described in methods section and the supplementary table. Materials availability is not applicable.

## Fundings

This work is supported by 10.13039/501100001809National Natural Science Foundation of China (grant number 82170882), Basic Research Project of Jiangsu Science and Technology Office (grant number BK20230075), a fellowship from 10.13039/501100002858China Postdoctoral Science Foundation (2023M731407), Jiangsu Provincial Medical Key Discipline (Laboratory) (ZDXK202202) and a Project Funded by the 10.13039/501100012246Priority Academic Program Development of Jiangsu Higher Education Institutions.

## Declaration of competing interest

The authors declare that they have no known competing financial interests or personal relationships that could have appeared to influence the work reported in this paper.
