# Supplementary material for: Evolutionary conservation analysis of human sphingomyelin metabolism pathway genes
Source: Heliyon. 2024 Nov 29;10(23):e40810. doi: 10.1016/j.heliyon.2024.e40810 (PMC11652929; doi:10.1016/j.heliyon.2024.e40810)
Supplement: Multimedia component 1 [file mmc1.docx]

Table S1. List of sphingomyelin metabolism pathway genes.

| Pathway | Gene symbol | Description |
| --- | --- | --- |
| *de novo* synthesis | SPTLC1 | serine palmitoyltransferase long chain base subunit 1 |
|  | SPTLC2 | serine palmitoyltransferase long chain base subunit 2 |
|  | SPTLC3 | serine palmitoyltransferase long chain base subunit 3 |
|  | KDSR | 3-dehydrosphinganine reductase |
|  | CERS1 | ceramide synthase 1 |
|  | CERS2 | ceramide synthase 2 |
|  | CERS3 | ceramide synthase 3 |
|  | CERS4 | ceramide synthase 4 |
|  | CERS5 | ceramide synthase 5 |
|  | CERS6 | ceramide synthase 6 |
|  | DEGS1 | delta 4-desaturase, sphingolipid 1 |
|  | DEGS2 | delta 4-desaturase, sphingolipid 2 |
| Salvage synthesis | SGPP1 | sphingosine-1-phosphate phosphatase 1 |
|  | SGPP2 | sphingosine-1-phosphate phosphatase 2 |
|  | GLB1 | galactosidase beta 1 |
|  | HEXA | hexosaminidase subunit alpha |
|  | HEXB | hexosaminidase subunit beta |
|  | NEU1 | neuraminidase 1 |
|  | NEU2 | neuraminidase 2 |
|  | NEU3 | neuraminidase 3 |
|  | NEU4 | neuraminidase 4 |
|  | GLA | galactosidase alpha |
|  | ARSA | arylsulfatase A |
|  | GBA1 | glucosylceramidase beta 1 |
|  | GBA2 | glucosylceramidase beta 2 |
|  | GALC | galactosylceramidase |
| Sphingomyelin synthesis | SGMS1 | sphingomyelin synthase 1 |
|  | SGMS2 | sphingomyelin synthase 2 |
| Sphingomyelin hydrolysis | SMPD1 | sphingomyelin phosphodiesterase 1 |
|  | SMPD2 | sphingomyelin phosphodiesterase 2 |
|  | SMPD3 | sphingomyelin phosphodiesterase 3 |
|  | SMPD4 | sphingomyelin phosphodiesterase 4 |
|  | ENPP7 | ectonucleotide pyrophosphatase/phosphodiesterase 7 |
| Other metabolic pathway | ASAH1 | N-acylsphingosine amidohydrolase 1 |
|  | ASAH2 | N-acylsphingosine amidohydrolase 2 |
|  | ACER1 | alkaline ceramidase 1 |
|  | ACER2 | alkaline ceramidase 2 |
|  | UGT8 | UDP glycosyltransferase 8 |
|  | UGCG | UDP-glucose ceramide glucosyltransferase |
|  | B4GALT5 | beta-1,4-galactosyltransferase 5 |
|  | B4GALT6 | beta-1,4-galactosyltransferase 6 |
|  | CERK | ceramide kinase |
|  | SPHK1 | sphingosine kinase 1 |
|  | SPHK2 | sphingosine kinase 2 |
